# Deadly Drugs and Curious Antidotes

**Published:** 1886-04

**Authors:** 


### DEADLY DRUGS AND THEIR CURIOUS ANTIDOTES.

Dr. Verne in a recent lecture on the subject of irritant poisons before a meeting of the Kings County Pharmaceutical Society said:

"The smallest amount of sulphuric acid known to have produced death is one drachm, and recoveries have been made after the patient has taken two ounces. Death may occur in a few hours or it may be delayed days and months if the acid has been mixed with food or swaetened in any other way. Sulphuric acid turns the æsopagus and tissues black, nitric acid makes them yellow and hydrochloric acid makes them at first white, then black, so that in making a diagnosis it is quite possible to confuse hydro-chloric with sulphuric acid. Nitric and hydro-chloric acids are of about equal strength. You get with them marked pain in the stomach, collapse, coldness, rapid perspiration and finally death. Sometimes, with them death is caused by shock as in cases where a limb is crushed or torn off by machinery. They coagulate the albumen of the tissue, oxidising it and producing the same effect as if it were destroyed by fire. Where they are more diluted they act as simple irritants. Now for the antidotes. An emetic would hardly act upon a stomach in which the tissue is destroyed, while a stomach pump may punch a hole, so to speak, through the weakened walls. Therefore you give the chemical antidote which is, as might naturally be expected, an alkali, yet you are not to use large quantities, as you use large quantities of belladonna to counteract the effects of opium, because the gases generated from the combinations formed when the alkali meets the acid would cause disastrous explosions. You give therefore a diluted alkali. Diluted solutions of soda or potash are good and soapsuds is very good and very accessible. The alkaline carbonates, lime, magnesia all well diluted are very serviceable. Then the patient must also be treated for collapse and must be stimulated. You can't use stimulant by the stomach because that is in no condition to dispose of it. Alcoholic injections are, therefore, to be recommended. Now we come to acetic and oxalic acids. Acetic acid is not often used and is not likely to be taken by mistake, as it is more pungent even than vinegar. The symptoms are the same as in cases of the stronger mineral acids. Oxalic acid is something very easily obtained. It is used to clean boilers, for instance, and as there are fashions in poisons as well as other things, it was once quite a fashionable poison for suicides, but has since been superceded by more popular drugs. It causes burning pain, nausea and depression, with not so much destruction of tissue as other poisons mentioned. In treating a patient for oxalic acid poisoning give chalk or carbonate of magnesia, but not the stronger alkalis, because they combine with it to form salts which are equally poisonous. The antidote for acetic acid is the same as for oxalic acid. The alkaline poisons with the same results as the acids act differently. They rapidly combine with the albuminous substances and burn the tissue, making it soft, flabby and swollen and easily broken down, the condition differing entirely from that resulting from the acids. Alkaline carbonates, such as carbonate of potash, may cause death in very much the same manner. Where the acid tends to confine itself the alkali tends to spread over the tissue. The symptoms are the same as those produced by acids with the exception of ammonia—pain, burning, depression, nausea and death. Ammonia adds the danger of suffocation to its other evils. Sometimes it is necessary to open the windpipe to relieve the patient. The antidotes are weak acids, vinegar being good and dilution of the vinegar being sometimes necessary. So much for the stronger corrosives.

Ten grains of sulphate of potash have been known to cause death and a dose of one and one-half ounces of cream of tartar has also resulted fatally. Lime is very rarely the cause of poisoning, though sometimes children try to eat slack lime and caustic corrosive poisoning ensues. Sulphate of zinc, chloride of zinc, nitrate of silver, sulphate of iron, chloride of tin, and bicromate of potash are all poisonous, the latter being powerful. They all give the same symptoms of corrosion, burning pain, thirst, vomiting, purging and even collapse and death. For lime, salts, mild acids and mucilaginous drinks are to be recommended, and in case of chloride or sulphate of zinc the same treatment is used to evacuate. For lime and silver combinations the same also, while protecting the stomach with milk or flour or starch, mucilage or flaxseed tea. The antidote for nitrate of silver poisoning is ordinary table salt. For the vegetable cathartics emulsive drinks to coat over the intestines should be used. Opium is the antidote, if it can be called an antidote; it soothes the pain and checks the rapid secretion which is proceeding. Men differ greatly in the quantities of iodine which they can take with safety, one person being able to stand ten times as large a dose as another. To some also iodide of potassium is very poisonous, while others can take sixty grains per day and feel no ill effects.

---

**Dio** *Lewis* Nuggets has been suspended. It was a most excellent publication and deserved success. People don't learn what is good for them easily. If it had some way of telling how to make ten dollars so that they could see the money they would all take it.

